# Carbon nanomaterials for drug delivery and tissue engineering

**DOI:** 10.3389/fchem.2022.990362

**Published:** 2022-09-12

**Authors:** Shaolie Zheng, Yuan Tian, Jiang Ouyang, Yuan Shen, Xiaoyu Wang, Jian Luan

**Affiliations:** ^1^ Department of Obstetrics and Gynecology, The First Affiliated Hospital of Jinan University, Guangzhou, China; ^2^ Department of Chemistry, Jinan University, Guangzhou, China; ^3^ College of Sciences, Northeastern University, Shenyang, China

**Keywords:** carbon dots, carbon nanotubes, graphene, drug delivery, tissue engineering

## Abstract

Carbon nanomaterials are some of the state-of-the-art materials used in drug-delivery and tissue-engineering research. Compared with traditional materials, carbon nanomaterials have the advantages of large specific surface areas and unique properties and are more suitable for use in drug delivery and tissue engineering after modification. Their characteristics, such as high drug loading and tissue loading, good biocompatibility, good targeting and long duration of action, indicate their great development potential for biomedical applications. In this paper, the synthesis and application of carbon dots (CDs), carbon nanotubes (CNTs) and graphene in drug delivery and tissue engineering are reviewed in detail. In this review, we discuss the current research focus and existing problems of carbon nanomaterials in order to provide a reference for the safe and effective application of carbon nanomaterials in drug delivery and tissue engineering.

## 1 Introduction

Cancer is a disease that greatly affects human survival in this century (Banerjee, 2010; Ouyang et al., 2018a; Ouyang et al., 2018b). Chemotherapy is, currently, one of the clinical methods for treating cancer, but it has serious side effects and limited and individually differenced therapeutic effects (Arias, 2011; Mohanty et al., 2011; Ouyang et al., 2022). To overcome the problems (at least some of them) of chemotherapy in cancer therapy strategies, over the past few decades, various drug-delivery systems have been explored with the aim of targeting cancer delivery and controlled release of therapeutic drugs within lesions (Hubbell and Chilkoti, 2012; Lee et al., 2014; Chen et al., 2017a; Ouyang et al., 2021). In particular, in order to improve the specificity of treatment, researchers have used nanotechnology to design different types of targeted drug-delivery systems, which have been proven effective in preclinical animal experiments (Jin et al., 2015; Zheng et al., 2015; Luo et al., 2016; Chen et al., 2017b). Targeted drug-delivery vehicles for cancer therapy not only exhibit excellent *in vivo* pharmacokinetic characteristics and tumor-homing ability but also enable selective control of drug release in diseased regions (e.g., cancer), resulting in the high efficacy and minimal side effects of cancer-specific treatments (Chen et al., 2014; Das et al., 2015; Wang et al., 2018).

Tissue engineering involves the reproduction and regeneration of damaged organs and tissues (Webber et al., 2015; Langer and Vacanti, 2016). Engineered tissues require tissue, cellular, morphological and physiological characteristics that are similar to those of *in vivo* tissues to achieve their potential (Freed et al., 1994; Khademhosseini et al., 2009; Tamayol et al., 2013). Therefore, the design of functional tissue engineering must possess several key elements for guiding cell growth and regulation, delivering bioactive molecules and generating appropriate physical and chemical signaling cues. These can be achieved by specific nanomaterials that possess the unique features and properties among single atoms and continuous bulk structure.

Carbon is one of the most important and closely related elements in nature. Carbon atoms can form stable single, double and triple bonds with sp^3^, sp^2^ and sp hybrid orbitals, respectively, thus forming a variety of functional materials with completely different structures and properties. For example, quaternized carbon nanospheres (QCNSs) (Jiang et al., 2017) with superior antibacterial activity; water-dispersible carbon nano-onion clusters (CNOCs) (Sun et al., 2019) with high photothermal conversion efficiency; and nano-diamonds (Gupta et al., 2017) that can be used to treat cancer, etc. In recent decades, the importance and potential of carbon nanomaterials have been recognized by some of the highest scientific awards, including the 1996 Nobel Prize in Chemistry (fullerenes), the 2008 Kavli Prize in Nanoscience (carbon nanotubes) and the 2010 Nobel Prize in Physics (graphene). These show that carbon nanomaterials and their applications are encountering their most rapid development and represent a very important topic in modern materials science (Lu and Schüth, 2006; Linares et al., 2014; Rondeau-Gagné and Morin, 2014; Titirici et al., 2015).

**Scheme 1:**
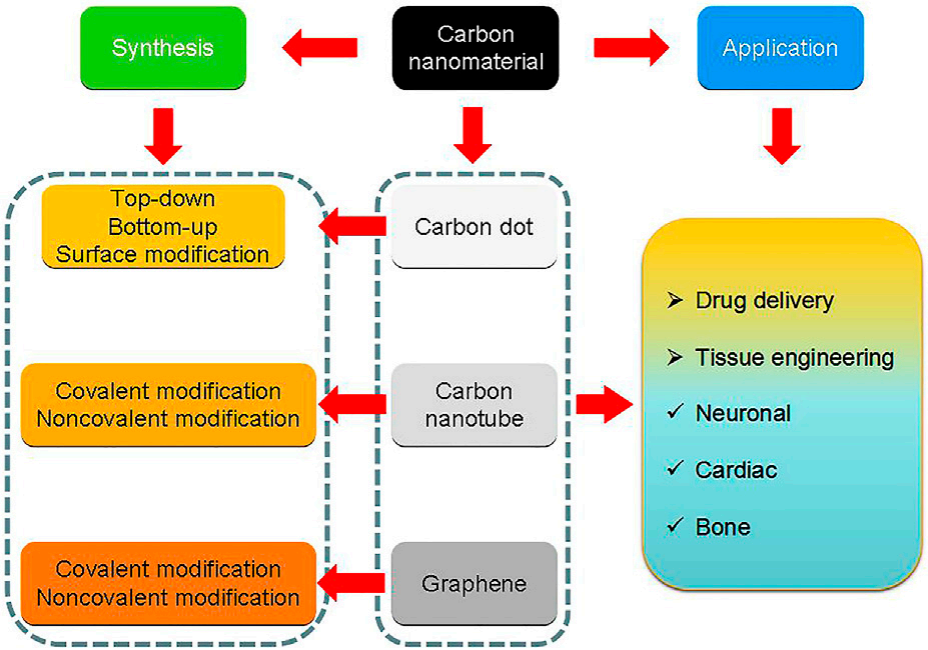
The outline in this review.

This article presents the latest trends in the synthesis, characterization and applications of carbon nanomaterials (such as CDs, CNTs, graphenes and their composites) through a comprehensive analysis of the creations of some of the most prominent contributors. We conclude that the sustainable development of carbon nanomaterials science can provide innovative solutions for a sustainable future and a clean environment (Scheme 1).

## 2 Synthesis methods and related structures of carbon nanomaterials

### 2.1 Synthesis methods and related structures of CDs

**FIGURE 1:**
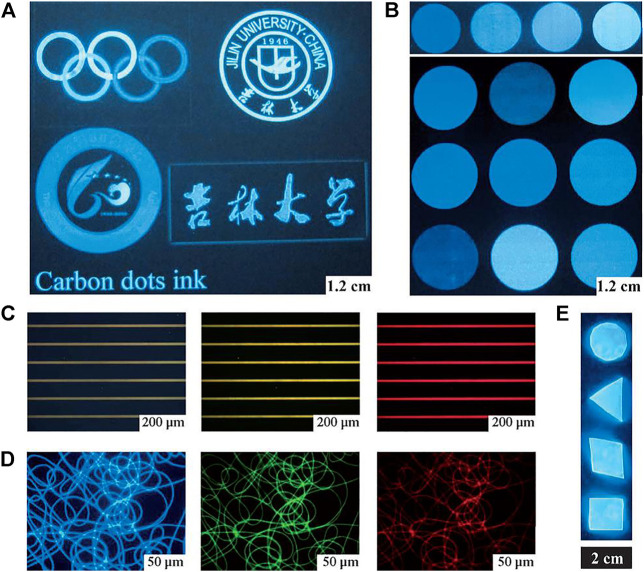
Printed patterns obtained by CD ink and the integration of CDs and polymers. **(A)** Different graphic patterns on paper (illuminated by a portable UV lamp). **(B)** Inks in multiple colors, tuned by the CD concentration in aqueous solution (illuminated by a portable UV lamp). **(C)** CD ink patterned on hydrophilic photoetching stripes (under UV, blue, and green light excitation). **(D)** Fluorescence microscopy images of PVA/CD nanofibers with UV, blue, and green light excitation. The fluorescence microscopy images in **(C)** and **(D)** were obtained through band-pass filters of different wavelengths: 450 nm, 550 nm, and 580 nm. **(E)** Bulk PDMAA/CD nanocomposites (The PL intensity was unchanged after 2,000 W UV exposure for 30 min).

Since 2006, CDs have received extensive attention as new members of the quantum dot family and are expected to become new fluorescent nanomaterials (Ponomarenko et al., 2008). CDs are a class of zero-dimensional (0D) carbon nanomaterials with a size of less than 10 nm and a 0.34 nm lattice corresponding to the (002) plane of graphene. When prepared using solution methods, CDs usually have many (epoxy, hydroxyl, carbonyl and carboxyl) functional groups on their surface. These functional groups lead to CDs being water-soluble and being able to connect to various organic polymers or biomolecules. The unique optical properties of tunable luminescence from the ultraviolet to the near-infrared (NIR) and multiphoton upconversion luminescence exhibited by CDs are influenced by surfaces, quantum confinement and edges. We, therefore, control these properties by controlling the shape and size of CDs, as well as by heteroatom doping and surface modification (Liu et al., 2013). In addition, compared with organic dyes and traditional semiconductor quantum dots, CDs not only have the characteristics of photobleaching resistance and photostability but also have better biocompatibility and lower toxicity. To reduce synthesis costs and take better advantage of the unique properties of CDs, researchers have carried out extensive research in a variety of fields, including optical sensing, biological imaging, photocatalysis and electrocatalysis. In 2013, researchers successfully produced fluorescent CDs with blue light. Their quantum yield (QY is about 80%) in aqueous solution is comparable to that of semiconductor quantum dots. This further promotes the development of CDs in the field of biological imaging (Figure 1) (Zhu et al., 2013). Since then, researchers have attempted to use CDs for biomedical and optoelectronics applications such as photodynamic therapy, drug delivery, solar cells and light-emitting diodes. In recent years, graphene quantum dots (GQDs) have attracted great attention as another 0D fluorescent carbon nanomaterial. GQDs are defined as nanographene sheets less than 10 nm in size. Compared with CDs, they are usually a single graphene layer; therefore, they have fewer atomic layers and better crystallinity.

There are two methods for CD synthesis: bottom-up and top-down methods. The former consists in the pyrolysis and carbonization of small organic molecules or the stepwise chemical fusion of small aromatic molecules, while the latter is achieved by chemically, electrochemically or physically decomposing larger carbon structures into smaller carbon structures. The selected synthetic material and synthesis methods usually determine the physicochemical properties of the synthesized carbon spots, such as oxygen/nitrogen content, size, crystallinity and fluorescence properties, including fluorescence quantum yield, colloidal stability and compatibility, etc.

#### 2.1.1 Top-down methods

The first discovered and reported fluorescent CDs were prepared using the top-down method. The method is employed in the presence of water vapor, using argon as a protective gas, and the laser ablation of graphite is performed to synthesize carbon dots after surface passivation and acid oxidation. Since then, researchers have developed top-down methods by reducing the structure of graphene, graphene or Go sheets, and CNTs to achieve a perfect sp^2^ hybrid structure. For the top-down approach, scientists have developed a number of methods to fracture carbon structures into CDs, such as arc discharge, laser etching, nano-photolithography, electrochemical oxidation, hydrothermal or solvothermal, microwave-assisted, ultrasound-assisted, chemical stripping and nitric/sulfuric acid oxidation. These synthetic methods are always complex and uncontrollable, with relatively low yields and quantum yields, and some are even environmentally harmful, making them unsuitable for the mass production of CDs with high fluorescent quantum yields.

#### 2.1.2 Bottom-up methods

In contrast, bottom-up methods typically use designed precursors and preparation methods to control CDs with good molecular weight and properties, shape and size. At the same time, the bottom-up method is low-cost and can mass-produce fluorescent CDs, which is the prerequisite to ensure the practical application of CDs. Hydrothermal treatment of citric acid or carbohydrates, laser irradiation of toluene, stepwise solution chemistry of benzene derivatives and precursor thermolysis have been used to successfully prepare carbon points. In addition to the above precursors, glycerol, amino acids, ascorbic acid and other molecules rich in carboxyl, hydroxyl and amino groups are suitable carbon precursors. They are dehydrated at high temperatures and further carbonized to form carbon spots.Yang and co-workers synthesized nitrogen-doped carbon sites with about 80% quantum yield *via* hydrothermal treatment of citric acid and ethylenediamine (Zhu et al., 2013). In addition, the luminescence color and quantum yield of CDs can be regulated by adjusting the amount of auxiliary inorganic substrates such as KH_2_PO_4_, NaOH and H_3_PO_4_, or the ratio of reagents. For example, Bhunia et al. (2013) used different carbohydrates and dehydrating agents as carbon precursors to synthesize visible-light-tunable high-fluorescence CDs at different temperatures. In addition, nucleation and growth kinetics were controlled by changing the dehydrating agent and reaction temperature. This synthesis method can control particle sizes of less than 10 nm and can synthesize CDs with adjustable emission and relatively high fluorescence quantum yield.

#### 2.1.3 Surface modification

Surface modification is a prerequisite for the further biomedical applications of CDs. Using different surfactants, the solubility of CDs in non-polar solvents can be enhanced, while their fluorescence properties can be adjusted. CDs with different groups on their surfaces can be synthesized to fulfill different functional requirements. Therefore, suitable biodegradable polymer precursors with different group components, such as amines, carboxylic acids and hydroxyl, can be selected to modify CDs. For example, the carboxyl groups on the CD surface make it hydrophilic during synthesis and can be mixed with polyethylene-glycol-containing amino groups to generate functionalized CDs. Thus, proper surface modification is beneficial to the further application and development of CDs in the biomedical field before biological application.

### 2.2 Synthesis methods and related structures of CNTs

Bare CNTs have a strong hydrophobic surface and are difficult to dissolve in water. For biomedical applications, surface functionalization of CNTs can improve the biocompatibility of CNTs and reduce their toxicity. Surface modification of CNTs mainly includes non-covalent modification and covalent modification. Non-covalent modification is to connect amphiphilic polymers to the surface of CNTs through hydrophobicity, while covalent modification is to connect hydrophilic molecules to the surface of CNTs through covalent bonds to increase their water solubility.

#### 2.2.1 Covalent modification of CNTs

Various covalent-bond modification methods have been used in the functionalization of CNTs, among which oxidation reaction is the most important modification method. The oxidation of CNTs mainly uses nitric acid as oxidant. In this oxidation process, carboxyl groups appear at the ends and defect positions of CNTs, which makes CNTs water-soluble to a certain extent. However, the oxidized CNTs accumulate and precipitate in salt solution, which cannot be directly used in living systems (because most living systems contain high-salt solution). Further surface modification is necessary for biomedical applications of CNTs. Polyethylene glycol (PEG), as a widely used surfactant, has been used to covalently modify CNTs *in vivo* and *in vitro*. Another widely used method of modifying CNTs is the cycloaddition reaction, which occurs on the side of the aromatic ring rather than at the end of the nanotubes or at the site of the defects formed by oxidation. The cycloaddition reaction connects the azide to the CNTs through a photochemical reaction. Prato et al. developed a method to modify CNTs by 1, 3-dipole cycloaddition reaction (Tagmatarchis and Prato, 2004). This method is also one of the most common modification methods.

Because the covalent modification method destroys the structure of carbon nanotubes, the inherent physical properties of carbon nanotubes, such as photoluminescence and Raman scattering, are destroyed after the chemical reaction. For example, the Raman scattering and photoluminescence intensity of single-walled carbon nanotubes (SWCNTs) are greatly weakened after covalent modification, which reduces the potential optical applications of this class of materials.

#### 2.2.2 Non-covalent modification of CNTs

Compared with covalent modification, the non-covalent modification of carbon nanotubes involves the modification of polymers or amphiphilic surfactant molecules onto the surface of CNTs. In the process of non-covalent modification, the chemical structure of CNTs is not damaged, but the length of CNTs is shortened by ultrasound in the process of functionalization, and other physical properties are retained. Therefore, the aqueous solution of CNTs, especially SWCNTs, can be used in various fields of biomedicine through non-covalent modification.

The aromatic carbon graphite surface of CNTs can be loaded with aromatic molecules by π–π stacking. Using the π–π interaction between CNTs and pyrene, Dai’s group used pyrene and its derivatives to non-covalently modify CNTs and found that proteins can be fixed to the CNTs modified by pyrene derivatives (Chen et al., 2001; Wu et al., 2008). In addition, Bertozzi et al. also modified CNTs with pyrene-linked sugar tree molecules (Moon et al., 2008). In addition to pyrene derivatives, single-stranded DNA is also widely used to modify carbon nanotubes, mainly through π–π stacking between the nanotube surfaces and the aromatic rings in DNA (Kam et al., 2005). However, Moon et al. found that the SWCNTs modified by DNA molecules were unstable *in vivo* because they contained nuclease to degrade DNA (Moon et al., 2008). Dai et al. found that fluorescein-labeled PEG chains could modify SWCNTs, and fluorescein labeled CNTs were obtained by π–π stacking between aromatic rings on fluorescein and CNTs for biological imaging (Nakayama-Ratchford et al., 2007). In addition, other aromatic molecules have also been used to non-covalently modify CNTs.

A variety of amphiphilic molecules are used to suspend CNTs by van der Waals forces and hydrophobicity to connect the hydrophobic part to the surface of the nanotubes, while the hydrophilic part is exposed to improve the water solubility of the nanotubes. In the process of biological detection based on SWCNTs, CNTs have been non-covalently modified with Tween-20 and block copolymer to reduce the non-specific adsorption between CNTs and proteins. Cherukuri et al. used block copolymer to modify CNTs for *in vivo* experiments (Cherukuri et al., 2006). However, using block copolymers to modify CNTs is not stable enough *in vivo* because proteins in plasma replace them. In addition, common surfactants such as sodium dodecyl sulfate and Tween-100 can be used to suspend CNTs, making them water-soluble. Amphiphilic-polymer-modified CNTs have a relatively high critical micelle concentration and are unstable in solution without the presence of other surfactants. This modification method is not suitable for use in biological systems, because large amounts of surfactant may dissolve cell membranes and denature proteins. An ideal non-covalent modification of CNTs for modifying biological systems should have the following characteristics: First, the modified molecule should be either non-toxic or biocompatible. Second, the polymer modified on the surface of CNTs should be stable enough to prevent dissociation in biological systems, especially in plasma solutions containing salts and proteins. Amphiphilic-polymer-modified CNTs should have a low critical micelle concentration value and maintain the stability of the CNTs after removing the excess modified molecules. Third, the modified molecule should have functional groups that can be used for biological coupling (such as linking antibodies or other molecules), allowing CNTs to be used for different aspects of biological applications.

**FIGURE 2:**
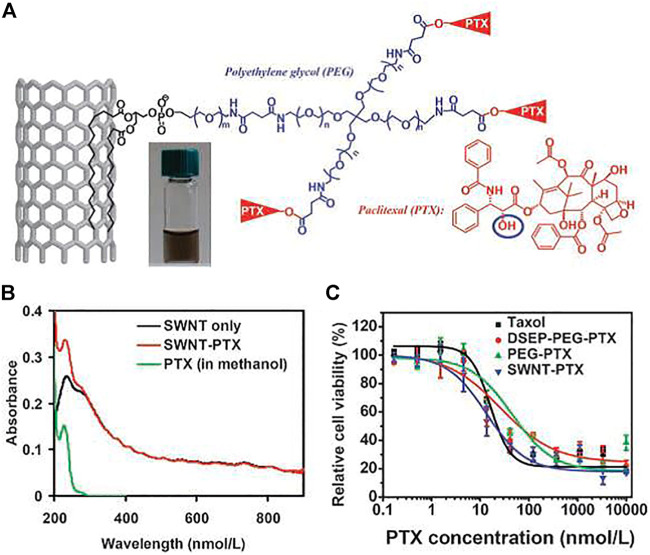
Carbon nanotube for PTX delivery. **(A)** Schematic illustration of PTX conjugation to SWCNT functionalized by phospholipids with branched PEG chains. **(B)** UV-VIS-NIR spectra of SWCNT before (black curve) and after PTX conjugation (red curve). **(C)** Cell survival versus concentration of PTX for 4T1 cells treated with Taxol, PEG-PTX, DSEP-PEG-PTX, or SWNT-PTX for 3 days.

Dai and co-workers developed non-covalent PEG-phospholipid (PL-PEG)-modified CNTs to meet the various needs of biomedicine and obtained highly water-soluble CNTs and their various functionalities (Figure 2) (Liu et al., 2008). Because phospholipids are a major component of cell membranes, they are safer than biological systems. During the modification process, two hydrocarbon chains of liposomes were anchored on the surface of the CNTs, while the hydrophilic chains extended to the water phase, making the CNTs water-soluble and biocompatible. Different from surfactant-modified CNTs, PL-PEG-modified SWCNTs have good stability in solution, including serum. PL-PEG of different PEG lengths and structures can be used to modify SWCNTs. Binding of biomolecules may be affected by amino interactions with PEG terminals. Using this strategy, PEG-modified single-walled CNTs have been successfully applied in the biomedical field, mainly including biological detection, biological imaging and drug delivery.

### 2.3 Synthesis methods and related structures of graphenes

The research upsurge of graphene has led to a strong interest in the preparation of materials by researchers at home and abroad, and different preparation methods are adopted according to their respective uses. Currently, graphene materials can be prepared by either bottom-up synthesis (including chemical vapor deposition (CVD) and chemical methods such as organic synthesis) or top-down synthesis (including mechanical stripping). In addition, the chemical oxidation method, the crystal epitaxy growth method and the CNT stripping method are also used (Loh et al., 2010; Yang et al., 2013a). Among these methods, the oxidative exfoliation method, which was discovered by Hummers and Offeman in 1958 to generate GO by reacting natural graphite with strong acids and strong oxidizing substances, is widely used to prepare graphene oxide (GO) (Hummers and Offeman, 1958). This method is the easiest way to obtain large quantities of nanographene and its derivatives. The prepared GO leaves a large number of epoxy, hydroxide and carboxyl groups on its surface for subsequent surface modification or linking to targeted molecules such as peptides or antibodies.

Many articles have reported the extensive application of GO and its derivatives (including reduction GO (RGO) and GO complexes) in biomedicine, but good surface modification of GO and its derivatives is essential. For example, Liu’s group prepared RGO with oxygen-containing hydrophilic groups, which were lost in the reduction process; it existed in the form of particles in aqueous solution and was not soluble in water. In addition, although the prepared GO can maintain good stability in aqueous solution, GO agglomerates in physiological solutions such as PBS, normal saline and cell culture medium, which may be caused by the charge-shielding effect in the presence of salt ions. Only through good surface modification can we improve the stability of nanomaterials, including GO *in vivo*, and control their behavior *in vivo*. GO and its derivatives can be modified appropriately according to different use purposes. At present, a variety of modification methods, including covalent modification and non-covalent modification, are widely used in the surface modification of GO and its derivatives for biomedical applications (Feng and Liu, 2011; Yang et al., 2012a). In addition, many inorganic nanoparticles are grown *in situ* or adsorbed onto the GO surface to obtain functional complexes based on nanographene (Yang et al., 2012b).

#### 2.3.1 Covalent modification of graphenes

Many active chemical groups (such as hydroxyl groups, epoxy and carboxyl) exposed at the GO edges and defect sites can be covalently linked to modify nanographene (Park and Ruoff, 2009). PEG is a strong hydrophilic polymer, which is widely used for modifying all kinds of nanometer materials, so as to improve their biocompatibility, reduce the nanomaterials non-specific adsorption of biological molecules and cells, and further improve the nanomaterials *in vivo* to better pharmacokinetically promote tumor targeting (Liu et al., 2007a). In 2008, Dai and co-workers used the amino-terminal-branched PEG to modify GO for the first time, mainly connecting the amino group of PEG to the carboxyl group on the surface of GO, so as to obtain PEG-modified GO (NGO-PEG) of ultra-small size (10–50 nm). NGO-PEG has shown excellent stability in a variety of physiological solutions (Yang et al., 2010). Since then, PEG-modified GO has been widely used in cell and *in vivo* biomedical research (Yang et al., 2010; Yang et al., 2011b)

In addition to PEG modification, many other hydrophilic macromolecules are used to covalently modify nanographene. Liu’s group covalently modified GO with aminated dextran, which could significantly improve the stability of GO in physiological solution (Zhang et al., 2011). Bao et al. used chitosan to covalently modify nanographene for drug and gene delivery (Bao et al., 2011). In addition, Gollavelli’s and Ling’s groups modified the GO functional complex with polyacrylic acid to improve its biocompatibility (Gollavelli and Ling, 2012). In addition to reacting with the carboxyl group on the GO surface, the epoxy group on the GO surface can also connect to the polymer to improve its stability. In a recent study, Niu et al. modified GO using an amino group on polylysine linked to an epoxide group on the surface of GO (Shan et al., 2009).

In addition, besides using hydrophilic polymers to modify GO, there are some other methods, such as using some small molecules to modify GO. Zhang and co-workers reported that sulfonic acid can covalently connect to the GO surface to improve the stability of GO in physiological solution (Zhang et al., 2010). Prato’s group modified GO with methylene nitride *via* a 1,3-dipole cycloaddition reaction (Quintana et al., 2010). However, the use of small molecule modifications of GO may require more testing in biological experiments to determine their biocompatibility.

#### 2.3.2 Non-covalent modification of graphenes

In addition to covalently modifying nanographene, nanographene can also be non-covalently modified with polymers or biological macromolecules through hydrophobic interactions, π–π stacking or electrostatic adsorption. GO can interact with surfactants and hydrophilic polymers to improve their water solubility. However, for biomedical applications, biocompatible-polymer-modified GO is better than small-molecule-surfactant-modified GO. Park et al. (2010) used the biocompatible molecule triton to modify reduced nanographene. Hu et al. (2012) used Pluronic F127 (PF127) to modify nanographene. The hydrophobic part of PF127 was connected to the GO surface through hydrophobic action, and the hydrophilic part extended into the solution, so as to prepare a very stable GO PF127 complex. Recently, Professor Dai Hongjie’s group prepared an ultra-small RGO, which was non-covalently modified by amphiphilic PEGylated polymer and attached with Arg-Gly-Asp (RGD) to improve its biocompatibility (Robinson et al., 2011). In recent work, Liu’s group used branched PEG to modify RGO and obtained RGO–PEG with very stable properties. RGO–PEG has an extremely long blood circulation time and can be used for photothermal therapy of tumors (Yang et al., 2012b).

The non-covalent modification methods listed above are based on the hydrophobic forces between hydrophilic polymers on the GO surface. Since the surface of GO has a strong negative charge, a polymer with positive charge can bind to the surface of GO through the effect of charge, so as to achieve the effect of modification. Liu’s group modified GO with a positively charged polymer, polythylene-imine (PEI), commonly used in gene transfection to obtain a very stable GO–PEI complex in physiological solution, while reducing the toxicity of PEI alone and improving the efficiency of GO-based gene transfection (Feng et al., 2011). In addition, Dong et al. (2011) used the same method to modify graphene nanoribbons to obtain PEI–NGR complexes for cell gene transfection and *in situ* microRNA detection. Misra’s group used the same approach for drug delivery. DOX-loaded GO was modified with positively charged folic-acid-linked chitosan to obtain DOX–GO–Chitosan–Folate nanocarriers for pH-controlled drug release (Depan et al., 2011).

**FIGURE 3:**
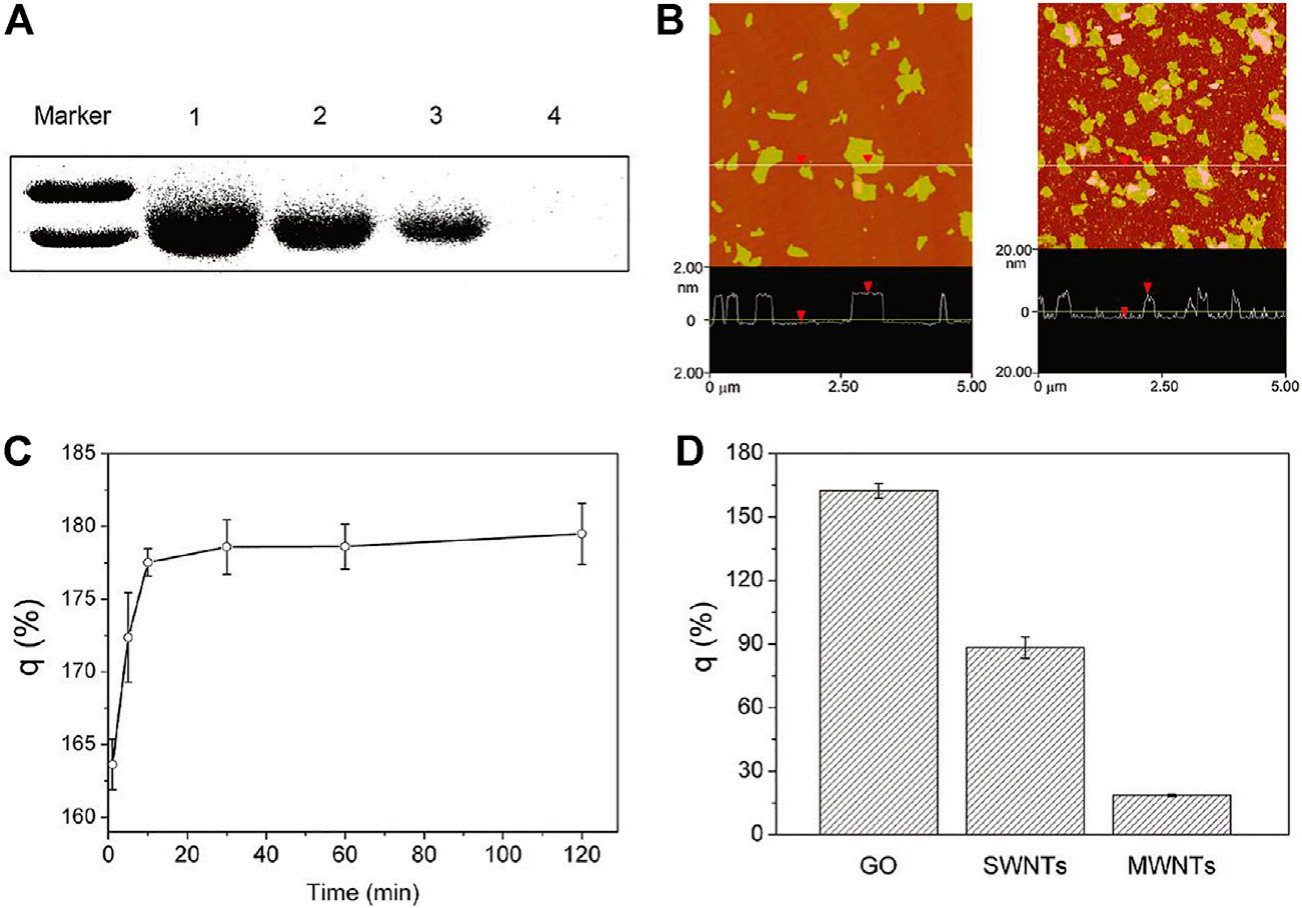
Characterization of interaction of GO with FBS proteins. **(A)** SDS-PAGE of FBS proteins in the supernatant after centrifugation. **(B)** AFM images of GO (left) and FBS-coated GO nanosheets (right). **(C)** FBS protein loading ratio on the surfaces of GO at different incubation times. **(D)** BSA loading capability of GO, SWCNTs, and MWCNTs.

In addition to the use of synthetic polymers to modify GO, some natural biomolecules such as proteins and DNA can also modify GO. Recently, Huang et al. modified GO with fetal bovine serum to obtain a GO–protein complex. Compared with GO without surface modification, GO–protein significantly reduced cytotoxicity (Figure 3) (Hu et al., 2011). In addition, non-polarized amino acids in BSA can also bind to the GO surface by hydrophobic forces. In another work, Liu et al. (2011) modified GO with gelatin. Graphene and GO have very high electrons on the surface, so GO can be linked to many aromatic molecules through π–π stacking.

## 3 Advances in the drug delivery of carbon nanomaterials

### 3.1 CD-based carbon nanomaterials for drug delivery

**FIGURE 4:**
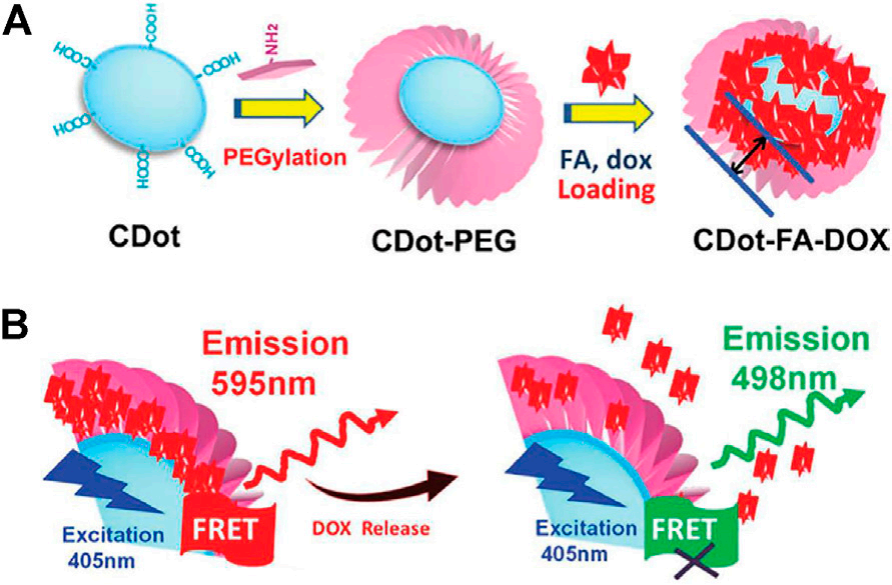
**(A)** A schematic representation of the surface coupling chemistry for FRET-CD-DDS. **(B)** Proposed mechanism of the FRET-CD-DDS for drug delivery.

Because CDs have many advantages, such as fast cell extraction, good biocompatibility, strong fluorescence, high stability and no influence on drug activity, many researchers use CDs as multifunctional carriers for drug release and loading (Wang et al., 2022). For example, CDs were embedded into the zeolite imidazole framework (ZIF), and the fluorescence intensity and size of the nanocomposite were optimized by changing the concentration of carbon points and precursors; then, 5-fluorouracil was loaded with the nanocomposite as a carrier for pH-responsive drug release. Tang et al. developed a fluorescence resonance energy transfer (FRET)-based carbon drug-delivery system (Figure 4) (Tang et al., 2013). CDs could serve as FRET donors or could be loaded with anticancer drugs. Since the distance between the acceptor and donor significantly affects FRET signaling, it can be used to sensitively monitor the release of the chemotherapeutic drug doxorubicin from carbon dots in real time. Hua et al. (2017).prepared a novel fluorescent carbon quantum dots (or carbon dots, CDs) for the first time by one-step hydrothermal treatment of chitosan, ethylenediamine and mercaptosuccinic acid. The as-prepared CDs have mitochondrial targeting ability without further modification of other mitochondriotropic ligands. The CDs-RB nanomissiles generated by conjugating CDs with the photosensitizer rose bengal (RB) can target/accumulate in the mitochondria of cells to achieve mitochondria-targeted photodynamic therapy. Yang et al. (2017). Passivated multifunctional carbon quantum dots (or carbon dots, CDs) with polyamine-containing organosilane molecules. The as-synthesized CDs exhibit negligible cytotoxic/systemic side effects, enabling simultaneous cellular imaging and anticancer drug delivery. Hua et al. (2018). Synthesized a novel class of multifunctional fluorescent carbon quantum dots (or carbon dots, CDs) by a one-pot hydrothermal reaction of m-phenylenediamine and L-cysteine, with no toxic side effects, can achieve high-quality nucleolar imaging in living cells and track nucleolus-related biological behaviors in real time. Furthermore, upon conjugation with the commonly used photosensitizer protoporphyrin IX (PpIX), the resulting CD-PpIX nanomissiles can rapidly and specifically target tumor sites and cause efficient tumor ablation after laser irradiation.Therefore, drug delivery based on CDs shows great potential in biomedical applications.

### 3.2 CNT-based carbon nanomaterials for drug delivery

Due to carbon nanotubes having different surface chemistries and sizes, they can enter cells through two pathways: a free diffusion/penetration pathway that requires no energy and an energy-dependent endocytosis pathway. Because all atoms are exposed on the SWCNT surface, it has a large specific surface area, so SWCNT can be loaded with proteins, DNA and aromatic drugs *via* π–π stacking and hydrophobic interactions (Nurunnabi et al., 2013; Yang et al., 2013b). In addition to non-covalent adsorption, small molecules of drugs can also be loaded onto carbon nanotubes through chemical bonds (such as ester bonds, disulfide bonds), giving these drugs highly stimulating and responsive drug release, thus achieving effective tumor therapy.

**FIGURE 5:**
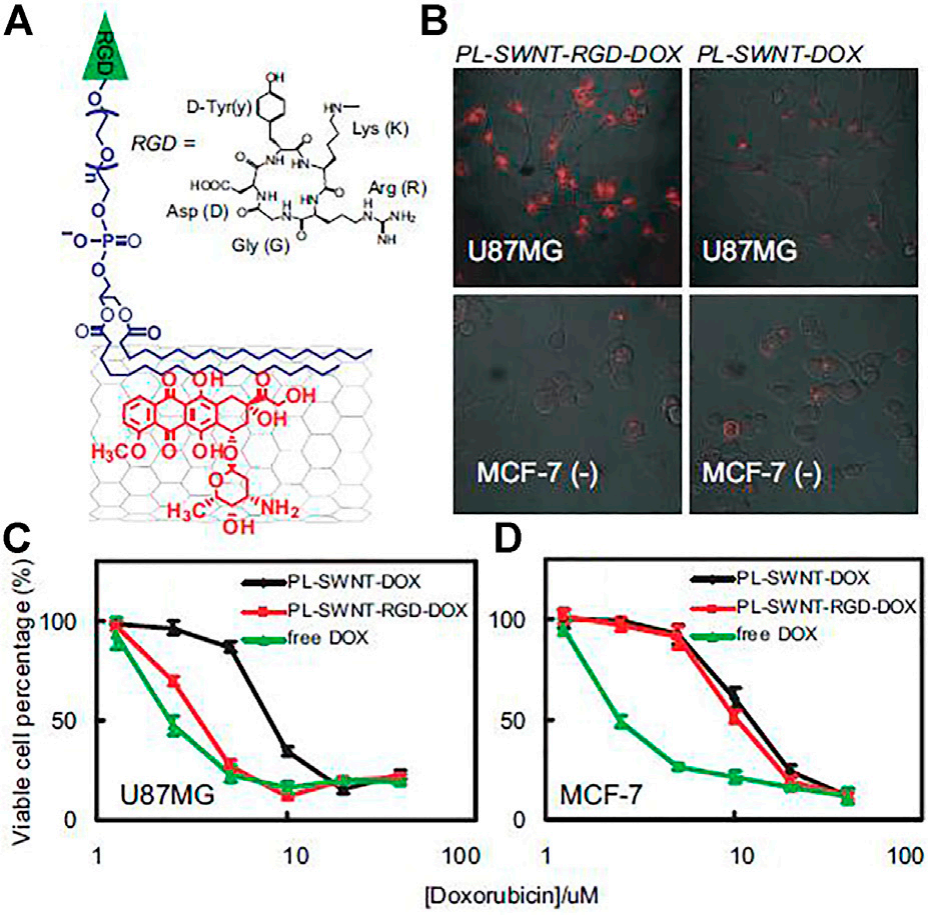
**(A)** Schematic structure of PLSWNT-RGD-DOX. **(B)** Confocal fluorescence images. Concentration dependent survival curves of U87MG cells **(C)** and MCF-7 cells **(D)** treated by various samples, as indicated.

In 2007, Dai’s group discovered that doxorubicin, widely used as an antitumor agent, could be effectively loaded onto the surface of SWCNTs (PL-PEG-modified) *via* hydrophobic interactions and π–π stacking. (Figure 5) (Liu et al., 2007b). The results showed that up to 4 g of DOX could be loaded per Gram of SWCNTs. DOX loaded onto SWCNTs can be used for pH-dependent drug release, and targeted cell killing can be achieved when targeted molecules are attached to the surface of PEG-modified SWCNTs (SWCNT-PEG). In subsequent work, DOX loaded on SWCNT-PEG was found to be less toxic to mice and to achieve good antitumor effects after intravenous injection into mice (Liu et al., 2009). Both studies demonstrated that suitably surface-modified SWCNTs would be good drug-delivery vectors. This efficient drug-loading strategy can be generalized to other structurally similar drug carriers (such as MWCNTs) and a variety of other aromatic drug molecules for effective drug loading and cancer treatment. Dahri et al. (2021) grafted a smart carrier composed of dimethylacrylamide-trimethyl chitosan (DMAA-TMC) on functionalized SWNTs, which improved the biocompatibility and biodegradability of the SWCNTs. Therefore, smart drug delivery carrier can be used to load, transport and release cancer chemotherapy drugs.

In addition, many studies have shown that some drug molecules can be linked to the functional groups or the surface-modified polymers on the surface of CNTs through breakable bonds. In 2008, Dai and co-workers connected paclitaxel (PTX; widely used as an anticancer drug) to a branched PEG (SWCNT–PEG–PTX) chain modified with SWCNTs *via* a broken bond. *In vivo* experiments showed that SWCNT–PEG–PTX had a good inhibitory effect on 4T1 breast cancer in mice, and the therapeutic results were better than those of paclitaxel (Liu et al., 2008). At the same time, platinum compounds (cisplatin prodrugs) were covalently linked to non-covalently modified SWCNTs such as paclitaxel (Dhar et al., 2008). They found that in the highly redox environment of cancer cells, conjugated platinum (IV) compounds could be easily reduced to cytotoxic cisplatin and specifically kill cancer cells with the help of folic acid linked to the surface of SWCNT–PEG. In addition to using SWCNTs as intracellular drug-molecule delivery carriers, MWCNTs are also effective drug carriers for drug delivery and tumor therapy (Wu et al., 2009).

### 3.3 Graphene-based carbon nanomaterials for drug delivery

In the past few decades, nanoparticle-based drug delivery has been widely used in tumor chemotherapy, with the aims of improving tumor treatment effectiveness and reducing toxic side effects. Since 2008, numerous research groups have been working on nanographene-based drug-delivery systems. Monolayers of GO or RGO can be used for drug loading due to their large specific surface area. The electrons on the nanographene surface can be combined with various aromatic drug molecules through π–π bonds; then, the functionalized GO or RGO surfaces can be linked with targeting molecules to selectively deliver drugs to specific cells.

Inspired by the use of CNTs for drug loading, GO with different surface modifications can also be used as a carrier to be connected with the loading of various anticancer drugs through physical adsorption or covalency. The anticancer drugs mainly include doxorubicin (DOX), camptothecin (CPT), SN38, ellagic acid, β-lapachone (β-lapachone) and 3-bis-(chloroethyl)-1-nitrosorea (BCNU). In 2008, Dai’s group used PEG-modified nanocrystalline graphene to obtain RGO–PEG, which is very stable in physiological solutions and can be loaded with the water-insoluble drug SN38 *via* π–π interactions. Compared with the anticancer efficiency of CPT11, the NGO–PEG–SN38 nanocomplex significantly improved its ability to kill tumor cells (Yang et al., 2010). The use of GO as a good drug carrier has also been reported by different research groups (Liu et al., 2011). To achieve targeted delivery of drugs into specific cells, Dai et al. attached an anti-CD20 antibody to the RGO-PEG surface and then loaded DOX to selectively kill B-cell lymphoma (Sun et al., 2008). Zhang et al. found that sulfonic-acid-modified GO coupled with folic acid could target cells with high expression of folate receptors. In addition, DOX and CPT, two anticancer drugs, were simultaneously loaded onto the GO surface to achieve the synergistic killing effect of tumor cells (Zhang et al., 2010).

**FIGURE 6:**
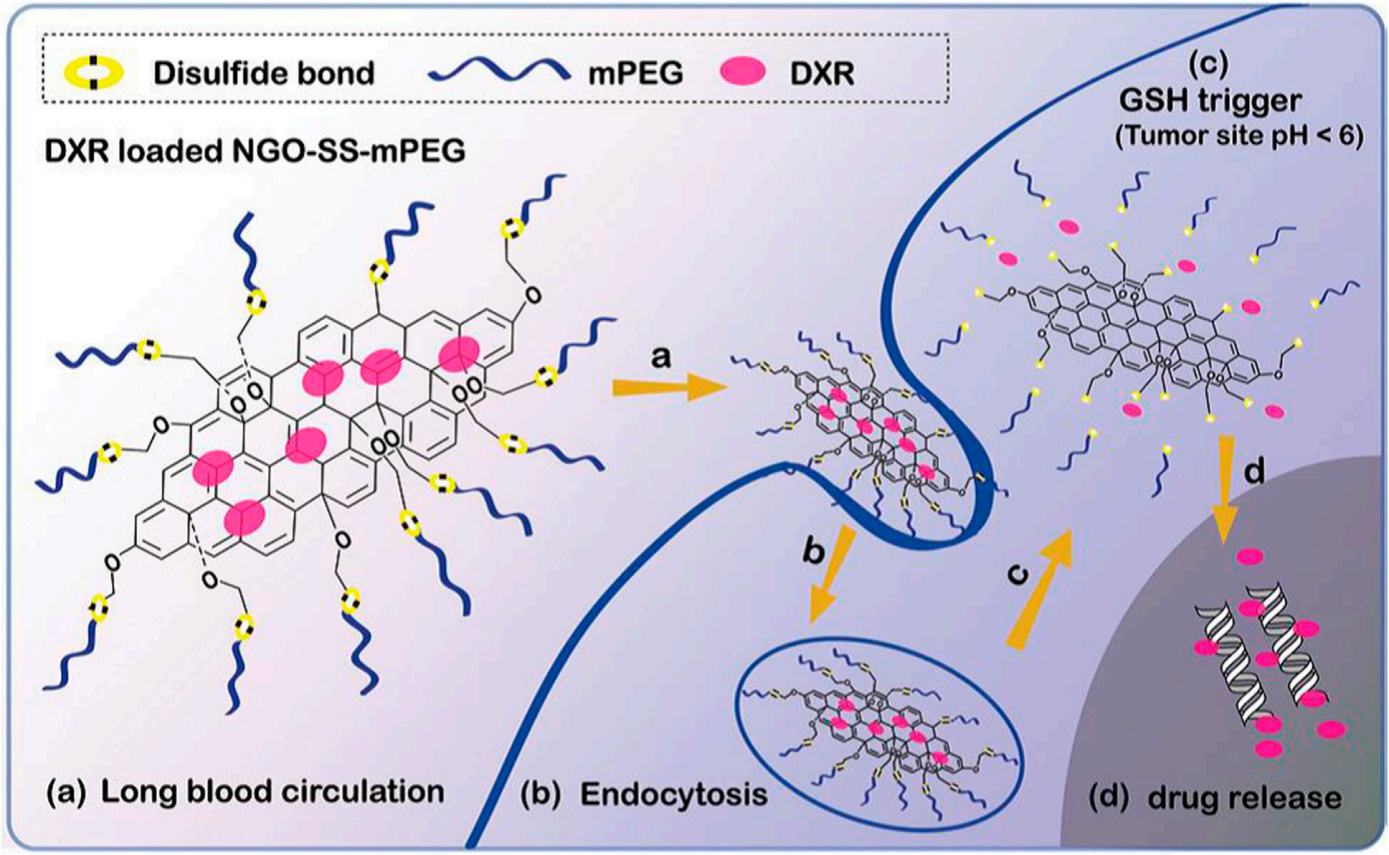
Schematic diagram showing antitumor activity of redox sensitive DXR-loaded NGO-SS-mPEG.

Recently, many research groups have also developed drug-delivery systems based on GO that can respond to environmental stimuli. Shi’s team developed GO modified with a PEG shell to prevent the NGO–PEG from releasing the loaded drugs (e.g., DOX). They modified GO with newly synthesized PEG cross-linked with disulfide bonds that release DOX upon cleavage of the disulfide bonds in a reducing environment and found that DOX delivery using such a loading system significantly improved the therapeutic efficacy of tumor cells (Figure 6) (Wen et al., 2012). In addition, Pan designed a heat-sensitive drug carrier based on GO. Firstly, the heat-sensitive polymer PNIPAM [Poly (N-isopropylacrylamide)] was chemically connected to the GO surface to obtain a very stable GO–PNIPAM complex under physiological conditions without obvious toxicity to cells. After loading CPT, GO–PNIPAM–CPT showed an excellent tumor-cell-killing ability compared with CPT alone (Pan et al., 2011).

In addition to using functionalized GO as a drug-delivery vector, many research groups still use drug delivery based on GO functional complexes. In 2009, Yang et al. (2009) used a GO–IONP nanocomposite to load DOX and achieve controllable release of DOX in pH response. At the same time, they utilized the magnetic properties of GO–IONP to link folic acid to achieve dual targeted drug delivery based on GO–IONP (Yang et al., 2011a; Yang et al., 2011b). Liu and co-workers prepared GO–IONP nanocomposites by high-temperature reactions and then covalently modified GO–IONP with amino PEG to improve its stability and biocompatibility. The GO–IONP–PEG obtained can be used for magnetic targeted drug delivery and photothermal therapy of tumor cells (Ma et al., 2012).

## 4 Advances in the tissue engineering of carbon nanomaterials

### 4.1 Application of CDs in tissue engineering

Manufactured scaffolds should possess the following properties to be applicable for tissue engineering: 1) high porosity of interconnected pore structure, 2) larger surface area, 3) suitable mechanical strength, 4) better biocompatibility and 5) controlled biodegradability (Nidhin et al., 2014; Raja and Fathima, 2018; Babbar et al., 2020). Meanwhile, CDs are integrated into various polymer scaffolds to enhance their application in tissue engineering (Shafiei et al., 2019).

#### 4.1.1 Cardiac-tissue engineering using CD-based carbon nanomaterials

**FIGURE 7:**
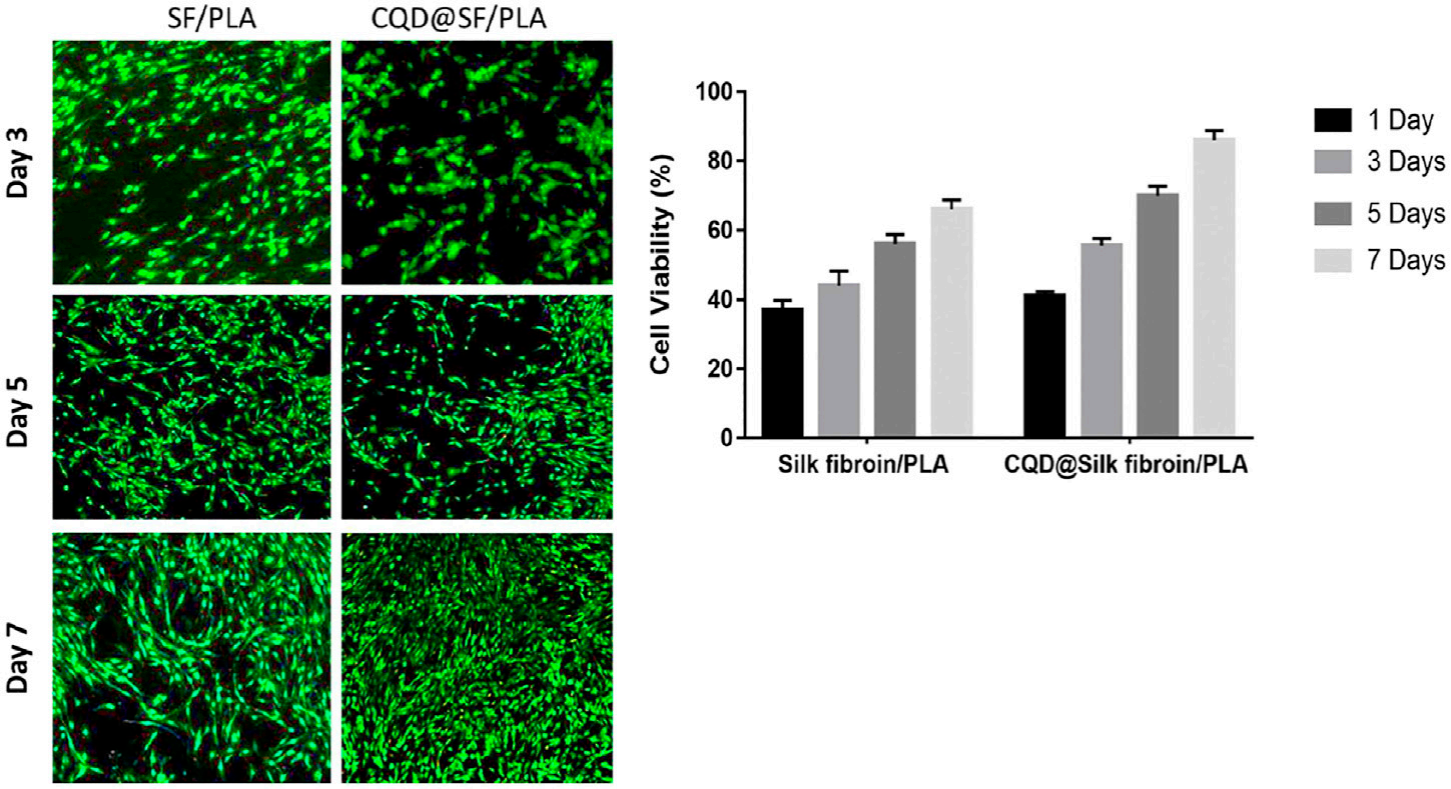
*In vitro* fluorescence cell viability images and Quantified cell viability of the Silk fibroin/PLA in the presence and absence of CQD nanoparticles at 1, 3, 5, and 7 days of culture.

Ren et al. prepared p-phenylenediamine functionalized CD-mediated silk fibroin-PLA (SF-PLA) nanofibrous scaffolds. It has better mechanical properties and can be applied in cardiac-tissue engineering and nursing care (Figure 7) (Yan et al., 2020). In the absence of any external electrical supply, the incorporation of CDs into SF-PLA scaffolds significantly enhanced cell adhesion, proliferation and mRNA expression of cardiac genes (Tnnc1, Tnnt2, Cx43, and Atp2a2).

#### 4.1.2 Bone-tissue engineering using CD-based carbon nanomaterials

**FIGURE 8:**
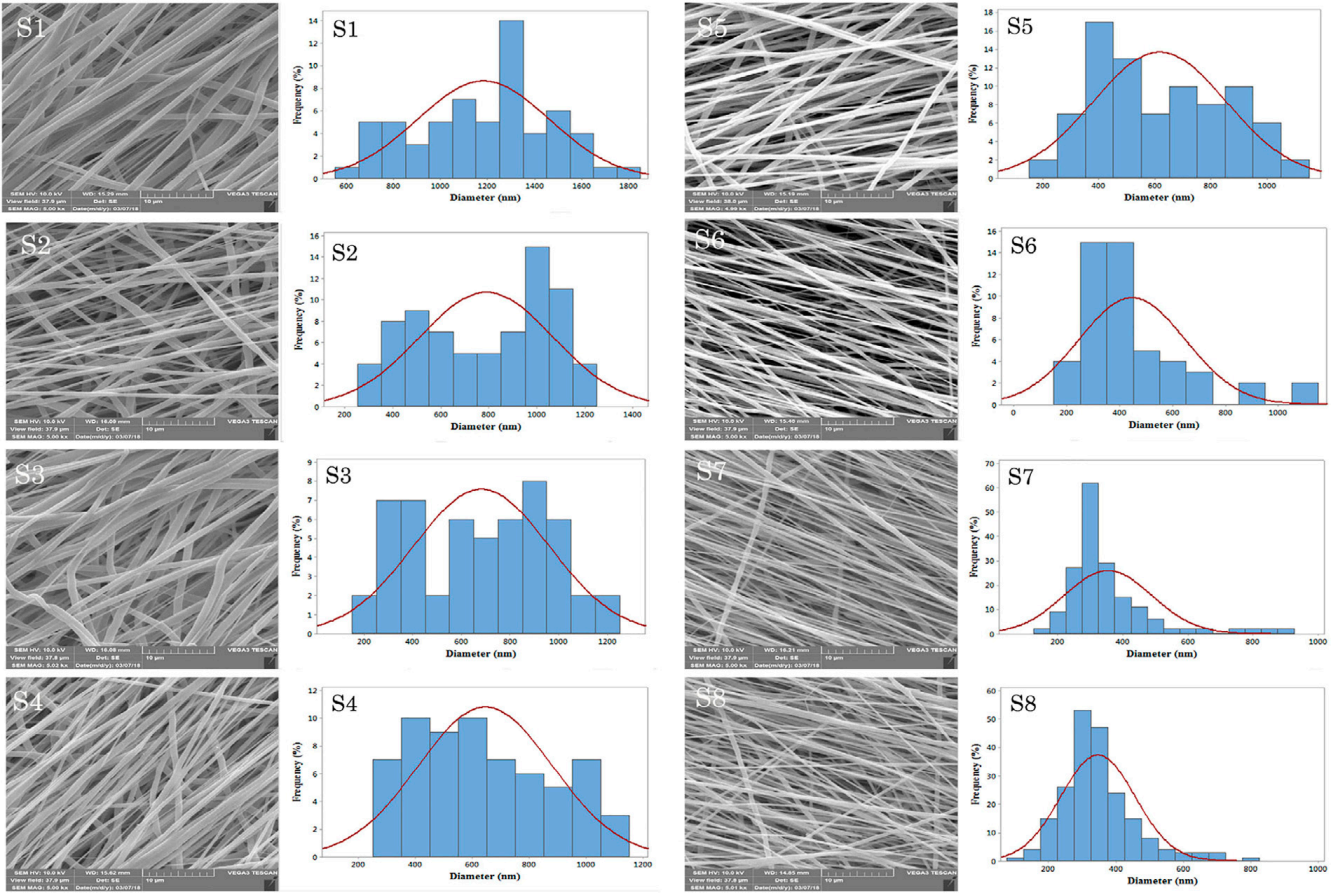
The SEM micrographs of electrospun scaffolds with the magnification of 5.00 Kx: S1, S2, S3, S4, S5, S6, S7, and S8.

Shafiei et al. developed nanofibrous scaffolds for efficient bone-tissue engineering application by an electrospinning method (Shafiei et al., 2019). Non-invasive scaffolds based on CD-peptide-mixed tannin-polyurethane (CDP-f-PU) fabricated by Gogoi et al. (2017) exhibited higher biocompatibility, osteoconductivity and osteodifferentiation ability in bone-tissue regeneration. Ghorghi et al. (2022) fabricated Captopril/CQDs/polycaprolactone (CP-CQDs-PCL) nanocomposite scaffolds with reduced fiber diameter due to the conductive properties of CQDs in the scaffolds (Figure 8). The scaffold with 0.5% CQD significantly increased the adhesion, proliferation and ALP activity of MG-63 cells, which was sufficient for bone-tissue engineering applications.

### 4.2 Applications of CNTs in tissue engineering

#### 4.2.1 Neuronal-tissue engineering using CNT-based carbon nanomaterials

It has been reported that CNTs can support neuronal attachment, promote the generation of neurites and be directly used as substrates for neuronal-tissue engineering (Koppes et al., 2016; Arslantunali et al., 2014; Lee et al., 2018; Shao et al., 2018; Shrestha et al., 2018; Wang et al., 2018; Wu et al., 2017; Xia et al., 2019). However, the potential toxicity of biological systems and the difficulty in generating canonical 2D/3D structures limit their utilization. Although polymer scaffolds have excellent biocompatibility, their poor electrical conductivity and the fact that they are not easily stretched limit their application in electrically propagating tissues. However, in combination with carbon nanotubes with strong electrical conductivity and good mechanical properties, they can be used in neuronal-tissue engineering, while the polymer scaffold minimizes the toxic effects of carbon nanotubes. Numerous studies have shown that carbon nanotubes and biocompatible polymers combined with each other can be used as two-dimensional conductive meshes or membranes for neural-tissue engineering (Table 1).

**TABLE 1:** A list of CNTs with polymers for neural tissue engineering.

No	CNT type	Polymer	Method	Reference
1	MWCNTs	Poly (dimethyldiallylammonium chloride)	Layer-by-layer assembly	Shao et al. (2018)
2	MWCNTs	Chitin	Solution and regenerating	Wu et al. (2017)
3	MWCNTs	Poly (2-hydroxyethyl methacrylate)	Polymerization process	Arslantunali et al. (2014)
4	MWCNTs	Hyperbranched polyglycerol sulfate	Dispersion and coating	Xia et al. (2019)
5	MWCNTs	PEGDA	Vat-photopolymerization	Lee et al. (2018)
6	MWCNTs	Chitosan/polyurethane/polypyrrole	Electrospinning	Shrestha et al. (2018)
7	MWCNTs	Poly (lactic-co-glycolic acid)	Electrospinning	Wang et al. (2018)
8	SWCNTs	Hydrogels	Gel method	Koppes et al. (2016)

Shao et al. (2018) synthesized membranes composed of carbon nanotubes and poly (Dimethyl Diallyl Ammonium chloride). These membranes can provide potent regulatory signals for the adhesion, differentiation and growth of neural-stem-cell-derived neurons. Wu et al. (2017) studies on Schwann cells showed that the biocompatible chitin/carbon nanotube (Ch/CNT) composite hydrogel not only had no cytotoxicity nor neurotoxicity but also exhibited increased proliferation, adhesion and diffusive bodies, and cells exhibited greater extension and elongation. Arslantunali et al. (2014) designed the MWCNT/poly (2-hydroxyethyl methacrylate) composite catheters, which can promote peripheral nerve regeneration.

More recently, Xia et al. (2019) reported electrospun fibrous scaffolds constructed using polyvalent polyanion-dispersed carbon nanotubes, which can be used for neural-tissue regeneration. The results demonstrated that the scaffold acts as a biocompatible platform for pluripotent stem cell (IPS) adhesion and proliferation, while being able to induce higher differentiation of IPSs. Furthermore, aligned fibers can guide neurites to grow in specific directions. Lee et al. (2018) synthesized MWCNT and PEGDA composite scaffolds; when combined with electrical stimulation, they showed synergistic effects on promoting nerve growth in neural-tissue engineering.

Shrestha et al. (2018) developed a self-electrically stimulating bilayer nerve guide catheter (NGC) for neural-tissue engineering. The results of cell experiments showed that this NGC can enhance the proliferation, differentiation and adhesion of PC 12 cells and Schwann cells. Furthermore, cells can be observed to distribute along the aligned fibers. Wang et al. (2018) produced a poly (lactic-co-glycolic acid) (PLGA) composite scaffold that could guide the growth of PC12 cells and DRG neurons along the fiber direction. Furthermore, by applying electrical stimulation, the material PC12 cells and DRG neurons loaded with polylactic-co-glycolic acid (PLGA) composite scaffolds exhibited longer neurite lengths, and PC12 cells under 40 mV electrical stimulation also increased compared with the control differentiation. Similarly, Koppes and colleagues (Koppes et al., 2016) synthesized composite scaffolds by mixing carboxylated SWCNTs with hydrogels, which could be applied by direct current stimulation. With the increase in SWNT loading, the electrical conductivity of the composite scaffolds increased significantly, resulting in a marked improvement in neurite outgrowth. Furthermore, by applying electrical stimulation, the growth of the SWCNT-loaded material was increased by a factor of 7.0 compared with the control. However, the exact mechanism of neurite elongation is not well understood, so further research is required.

#### 4.2.2 Cardiac-tissue engineering using CNT-based carbon nanomaterials

Biomaterials with sufficient mechanical strength and good electrical conductivity can be used in cardiac-tissue engineering. Despite their biocompatibility, polymeric materials are generally inert and require further improvement of their mechanical properties, which limit cell-to-cell interactions and signaling (Shin et al., 2013b; Kharaziha et al., 2014; Pok et al., 2014; Wickham et al., 2014; Sun et al., 2015; Liu et al., 2016; Yu et al., 2017; Mombini et al., 2019). Various polymer/CNTs composites with high mechanical and electrical properties of biopolymers have been investigated (Table 2).

**TABLE 2:** A list of CNTs with polymers for cardiac tissue engineering.

No	CNT type	Polymer	Method	Reference
1	MWCNTs	Gelatin methacrylate	Photo-crosslinking	Shin et al. (2013b)
2	MWCNTs	Collagen type I	Sonicated	Yu et al. (2017)
3	MWCNTs	Thiophene	Electrospinning	Wickham et al. (2014)
4	MWCNTs	Poly (lactic-co-glycolic acid)	Electrospinning	Liu et al. (2016)
5	MWCNTs	Poly (glycerisebacate)/gelatin	Electrospinning	Kharaziha et al. (2014)
6	SWCNTs	Gelatin chitosan hydrogel	Gel method	Pok et al. (2014)
7	SWCNTs	Collagen patch	Intercalated disc assembly	Sun et al. (2015)
8	SWCNTs	Polyvinyl alcohol/chitosan	Electrospinning	Mombini et al. (2019)

Shin et al. (2013a) prepared CNT–GelMA and cultured it with neonatal rat cardiomyocytes. Compared with controls, cardiomyocytes exhibited a 3-fold increase in spontaneous beating rate, an 85% lower excitation threshold and significantly improved cell adhesion. The experimental results obtained by Yu et al. (2017) showed that CNT–collagen scaffolds increased the rhythmic contractile area of cardiomyocytes (CMs), indicating improved function of CMs.

Wickham et al. (2014) showed that PCL and PCL/T-CNTs membranes did not improve the attachment and proliferation of cardiac stem progenitor cells (CPCs). In the case of electrospun meshes, the incorporation of T-CNTs can be observed to increase mechanical strength and cell proliferation, but no effects on cell differentiation are observed. Similarly, Liu et al. (2016) showed that electrospun poly (lactic-co-glycolic acid)/MWCNTs induce elongation of neonatal rat cardiomyocytes while enhancing the production of sarcomeric α-actin and troponin I in cardiomyocytes. The application of carbon nanotubes helps to overcome the limitation of insufficient remodeling of intercalated discs (IDs) connecting cardiomyocytes. Kharaziha et al. (2014) produced grids that were able to support cardiomyocyte ingrowth. Compared with the grids without MWCNTs, the grids containing MWCNTs exhibited stronger spontaneous and synchronous beating behavior, with a 3.5-fold lower excitation threshold and a 2.8-fold higher maximum trapping rate.

Pok et al. (2014) studies have shown that composite hydrogels can achieve excitation conduction velocities (22 ± 9 cm/s) similar to native myocardial tissue to support the function of cardiomyocytes. Therefore, they can be used as electrical nanobridges for communication between cardiomyocytes. Sun et al. (2015) developed a CNT/collagen patch that enhanced the assembly of intercalated discs in cardiomyocytes. Mombini et al. (2019) produced electrospun cardiac conductive scaffolds composed of chitosan (CS), polyvinyl alcohol (PVA) and carbon nanotubes at different concentrations (1, 3, and 5 wt%). Biological results showed that the electrical stimulation of scaffolds containing 1 wt% CNTs promoted gene expression of cardiac markers.

#### 4.2.3 Bone-tissue engineering using CNT-based carbon nanomaterials

Engineered tissue is used to produce synthetic 3D structural bone scaffolds that provide support for cell differentiation, attachment and proliferation (Gupta et al., 2014; Oyefusi et al., 2014; Duan et al., 2015; Khalid et al., 2015; Gonçalves et al., 2016; Tanaka et al., 2017a; Tanaka et al., 2017b; Jing et al., 2017; Wang et al., 2019; Świętek et al., 2019). Bone generates an electrical current when pressure is applied, so bone is a tissue with piezoelectric properties, whereas piezoelectric materials exhibit the reverse piezoelectric effect (The material compresses when an electrical current is applied) (Table 3); therefore, bone-tissue regeneration can be accelerated by using electroactive scaffolds and applying electrical stimulation.

**TABLE 3:** A list of CNTs with polymers for bone tissue engineering.

No	CNT tpye	Polymer	Method	Reference
1	MWCNTs	—	—	Tanaka et al. (2017a)
2	MWCNTs	—	—	Tanaka et al. (2017b)
3	MWCNTs	—	—	Oyefusi et al. (2014)
4	MWCNTs	—	—	Khalid et al. (2015)
5	MWCNTs	Poly (L-lactic-acid)	Freeze drying	Duan et al. (2015)
6	MWCNTs	Collagen/hydroxyapatite	Freeze-drying	Jing et al. (2017)
7	MWCNTs	PCL-hydroxyapatite	Extrusion-based additive manufacturing system	Gonçalves et al. (2016)
8	MWCNTs	Polymethyl methacrylate	Injection moulding	Wang et al. (2019)
9	MWCNTs	Polycaprolactone	Co-precipitation method	Świętek et al. (2019)
10	SWCNTs	Polylactic-co-glycolic acid	Oil-in-water emulsion method	Gupta et al. (2014)

Tanaka et al. (2017a) fabricated CNT-containing 3D bulk structures and investigated their efficiency as scaffolds for bone repair. Mechanical tests showed that the compressive strengths of the fabricated structures and rat femurs were 62.1 and 61.86 MPa, respectively, with no significant differences. In the presence of recombinant human BMP-2, CNT blocks showed higher ALP activity favoring osteogenic behavior. Tanaka et al. (2017b) concluded that carbon nanotubes have better protein uptake and release capacity than hydroxyapatite (HA). CNT porous structures combined with recombinant human BMP-2 showed higher cell proliferation, better osteoconductivity and more osteogenesis. Oyefusi et al. (2014) grafted HA onto carbon nanotubes and graphene nanosheets and then used human fetal osteoblast cell lines to study their effects on cell proliferation and differentiation. The experimental results showed that both CNTs–HA and graphene--HA materials can promote cell growth and differentiation. Khalid et al. (2015) synthesized MWCNT/HA scaffolds containing different loadings of CNTs (1 wt%, 3 wt%, and 5 wt%). The cytotoxicity of the composite scaffolds was dose-dependent with the CNT content. Experiments using a human osteosarcoma cell line showed that cell viability decreased with the increase in CNT content.

Duan et al. (2015) fabricated poly (L-lactic acid) (PLLA)/CNTs scaffolds using a freeze-drying method. *In vitro* studies have shown that CNTs can promote the cell proliferation and osteogenic differentiation of bone-marrow mesenchymal stem cells (BMSCs). *In vivo* experiments showed that CNTs significantly promoted the expression of osteogenesis-related proteins and the formation of type I collagen. Jing et al. (2017) reported that MWCNT scaffolds could enhance the proliferation rate of BMSCs and their protein expression of bone sialoprotein (BSP) and osteocalcin (OCN).

Goncalves et al. (2016) synthesized 3D-printed porous CNT scaffolds, which can be used for bone-tissue engineering. Cell proliferation experiments (6 days) were performed with MG63 osteoblast-like cells, and the results showed that the use of scaffolds containing 10 wt% MWCNTs had the most positive effect on cell proliferation.

Recently, Wang et al. (2019) introduced a polymethylmethacrylate (PMMA) bone cement loaded with different MWCNTs (0.1, 0.25, 0.5, and 1 wt%) that could be injection-molded for high-load joint replacement. *In vitro* studies have shown that this bone cement can promote the adhesion, proliferation and osteogenic differentiation of rat bone-marrow mesenchymal stem cells (rBMSCs). *In vivo* experiments have shown that PMMA bone cement containing 1 wt% MWCNTs could significantly enhance bone ingrowth, and the ingrowth rate was as high as 42% 12 weeks after surgery. Swietek et al. (2019) reported a solvent-casting method to combine functionalized CNTs (fCNTs) and iron oxide (ION) in two different mass ratios (1:1 and 1:4) to synthesize PCL-based 3D composite scaffolds for in-bone regeneration. The toxicity of composite scaffolds was assessed using the SAOS-2 human cell line. The results showed that fCNTs containing 1 wt% significantly improved cell attachment, and ion concentrations below 1 wt% increased cellular metabolic activity, indicating that the composite scaffold could be used for bone-tissue regeneration.

Gupta et al. (2014) fabricated SWCNT composite microspheres and polylactic-co-glycolic acid (PLAGA) for bone-tissue engineering. Compared with pure PLAGA scaffolds, MC3T3-E1 cells added SWCNT showed no abnormality in adhesion and growth, but the cell proliferation rate and gene expression were enhanced. The composites were further implanted in Sprague Dawley rats for 2, 4, 8, and 12 weeks. The results showed that the SWCNT/PLAGA composite has good *in vivo* biocompatibility. However, possibly due to the lack of a controlled porous structure, the material did not significantly improve bone-tissue repair.

### 4.3 Application of graphene in tissue engineering

Inspired by the mechanical, electrical and optical properties of nanographene, many research groups have developed potential applications based on nanographene substrates in tissue engineering. Therefore, it is increasingly important to understand the interaction between the nanographene matrix and cells.

#### 4.3.1 Neuronal-tissue engineering using graphene-based carbon nanomaterials

Charge-conducting polymers and carbon-based materials make biological interfaces conductive (Huang et al., 2013; Sinclair et al., 2013; Tu et al., 2013; Serrano et al., 2014; Song et al., 2014; Walters, 2014; Munoz et al., 2015; Pascual et al., 2015; Zhang et al., 2016). Among these conductive materials, graphene composites with excellent chemical, electrical and thermal properties and extremely low cytotoxicity are ideal materials for tissue engineering and prosthetics. Graphene and its derivatives are often used as neural structures in combination with neurotransmitters, anticoagulants and growth factors (Table 4).

**TABLE 4:** A list of graphenes with polymers for neuronal tissue engineering.

No	Graphene type	Polymer	Method	Reference
1	3D free standing porous and flexible GO	—	—	Serrano et al. (2014)
2	3D graphene foams	—	—	Song et al. (2014)
3	GO	PLLA nanofibrous	Coating	Zhang et al. (2016)
4	biomimetic GO	Dimethylaminoethyl methacrylate	Covalently linking	Tu et al. (2013)
5	biomimetic GO	MPC	Covalently linking	Tu et al. (2013)

Serrano et al. (2014) synthesized 3D free-standing porous and flexible GO scaffolds for nerve regeneration. Cell cultures showed that highly interconnected and viable neural networks formed on these scaffolds for 14 days. However, there is a lack of *in vivo* studies confirming that these scaffolds can guide nerve regeneration. Song et al. (2014) observed that 3D graphene had good biocompatibility and was able to promote the growth of microglia.

After coating the aligned PLLA nanofiber scaffolds with GO in the presence of the nerve growth factor (NGF), their hydrophilicity and surface roughness were significantly improved. This fibrous scaffold promotes the proliferation and differentiation of Schwann cells and rat pheochromocytoma 12 (PC12) cells (Zhang et al., 2016). Tu et al. (2013) constructed intelligent biomimetic GO-based composites. The acetylcholine-like unit (dimethyl aminoethyl methacrylate) and the phosphorylcholine-like unit MPC on the surface of GO can promote the germination and growth of neurites after being covalently linked together.

Neural-tissue engineering has also been developing materials that can induce neuroinflammation (Sinclair et al., 2013). Astrocytes, peripheral macrophages and microglia in the brain mediate neuroinflammatory responses to hypoxia, ischemia and viral and bacterial infections (Huang et al., 2013; Walters, 2014; Munoz et al., 2015; Pascual et al., 2015). Observations suggested that the neuroinflammatory response of 3D graphene to microglia is milder than that of 2D graphene, suggesting that topographical features may influence inflammatory behavior. Furthermore, 3D graphene foam promoted the growth of neural stem cells and PC-12 cells (derived from the neural crest), demonstrating their use in neural repair and neurogenesis.

#### 4.3.2 Cardiac-tissue engineering using CNT-based carbon nanomaterials

In cardiac-tissue engineering, graphene is often used to treat cardiovascular disease and prevent blood clotting in artificial heart valves (Ong et al., 2015; Sergi et al., 2015; Weijmans et al., 2015). Cardiovascular disease affects human health due to its high incidence and prevalence and is intensely studied in regenerative medicine (Shi et al., 2010; Şenel et al., 2014). The scarcity of donors and the high number of complications limit the use of heart transplantation for the treatment of cardiovascular disease (Shin et al., 2013b; Gálvez-Montón et al., 2013). The heart has dynamic functional characteristics and a complex tissue structure. Therefore, in cardiac-tissue engineering, an ideal artificial cardiac scaffold needs to be similar to the heart in structure, electrophysiology and mechanical properties to stimulate angiogenesis and maintain the viability of transplanted cells (Shin et al., 2013a; Radhakrishnan et al., 2014).

GO/gelatin methacrylate (GM) hydrogel is a synthetic cardiac scaffold that can be used to treat cardiovascular disease (Nguyen et al., 2015) GM facilitates the uniform distribution of GO in the hydrogel matrix and is a biocompatible surfactant. GO/GM hydrogels support cell spreading and alignment and enhance viability and proliferation in a 3D environment. Their tunable mechanical stiffness and electrical conductivity enable engineered cardiac patches to treat myocardial infarction (Kim et al., 2013; Kim and Song, 2014; Nguyen et al., 2015). Hydrogels can increase the local retention time of the carriers at the target site and increase their likelihood of being internalized by the tissue (Bae et al., 2012; Qi et al., 2014). Studies have reported that PLL can enhance the biological activity of GO in tissue engineering. GO/PLL composites have strong biocompatibility and high mechanical properties, which can accelerate cell growth and differentiation (Shin et al., 2014).

#### 4.3.3 Bone-tissue engineering using CNT-based carbon nanomaterials

Graphene is a multifunctional carbon-based material that can serve as a framework for multicomponent nanostructured biomimetic scaffolds with bone-like morphology and chemical structure (Deepachitra et al., 2013; Lalwani et al., 2013; Depan et al., 2014; Oyefusi et al., 2014). The application of graphene nanocomposites containing polymers (chitosan, collagen, polypropylene fumarate) in bone-tissue engineering is discussed in detail in the following sections (Table 5).

**TABLE 5:** A list of graphenes with polymers for bone tissue engineering.

No	Graphene tpye	Polymer	Method	Reference
1	Polyethylene glycol-functionalized GO	Poly (propylene fumarate)	Freeze-drying	Depan et al. (2014)
2	GO	Chitosan/HPA	Chemical conjugation	Oyefusi et al. (2014)
3	Multi-walled GO nanoribbons or GO nanoplatelets	Polypropylene fumarate	Thermal cross-linking and specimen fabrication	Lalwani et al. (2013)
4	Surface functionalized GO	Fibrin	Coating	Deepachitra et al. (2013)

GO is uniformly dispersed in the matrix, generates strong interfacial adhesion through polar and hydrogen bonding interactions and provides sufficient stiffness and strength in the biological state, providing effective support for the formation of bone tissue (Depan et al., 2014). Recently, a research group has synthesized a layered hybrid system composed of chitosan, GO and HPA (Oyefusi et al., 2014). The GO layer provides biocompatibility and mechanical strength; the HPA layer provides higher bioactivity; and chitosan in the middle layer further strengthens the hybrid material. Incubation of pre-osteoblasts (MC3T3-E1) with this hybrid system promotes uniform cell mineralization, proliferation and enhancement of proteins (actin, vinculin and fibronectin). These properties are important for cell adhesion and osteoblast morphogenesis (Oyefusi et al., 2014). Lalwani et al. (2013) synthesized PPF 2D nanocomposites containing GO nanosheets, and MoS2 nanosheets showed the highest mechanical strength and were ideal candidates for BTE. Deepachitra et al. (2103) studies on osteosarcoma bone cell lines (SaOS-2, CPC-2, MG-63, etc.) showed that fibrin is an important marker of osteoblast differentiation. Colorimetric assays (MTT, alkaline phosphatase (ALP) and *in vitro* calcium release) have shown that fibrin-modified GO has good biocompatibility and enhances osteoconductivity, making it an ideal scaffold for BTE.

## 5 Conclusion and outlook

In this review, we summarize recent advances in the preparation and application of carbon nanomaterials for drug delivery and tissue engineering. Such materials have excellent chemical, optical and mechanical properties. Although these nanomaterials are all made of the same carbon element, allotropes are made with different carbons and have different properties. Such materials also behave differently in the body depending on how carbon atoms combine at the nanoscale to form large nanostructures. Using mature surface modification methods, carbon nanomaterials with a good water solubility and biocompatibility can be obtained for use in drug delivery and tissue engineering.

The uniqueness of carbon nanomaterials provides many new approaches and opportunities for future applications. Cutting-edge research based on the intersection of carbon nanotechnology and biomedicine has demonstrated the promise of the use of various carbon nanomaterials for various applications, especially some very unusual properties and capabilities of carbon materials that are not available in other nanomaterials. NIR-II fluorescence imaging based on SWCNTs has changed the characteristics of the low penetration depth and sensitivity of conventional fluorescence imaging, and this technology is a new field of development. In the past few years, we have also witnessed the application of imaging techniques based on SWCNTs for NIR-II fluorescence imaging with a high sensitivity, low toxicity and deeper level of penetration. The high loading capacity of carbon nanomaterials, especially graphene and its derivatives, enables the efficient loading of drug molecules/nanoparticles by π–π stacking. However, based on the clinical application of carbon nanomaterials in the future, we still have some problems to think about and solve:

1 What is the future development direction of drug delivery and tissue engineering based on carbon nanomaterials?
2 What are the major obstacles we need to overcome before carbon nanomaterials can be used in clinical applications?
3 Can carbon nanomaterial-based drug delivery and tissue engineering inspire us to explore new directions in chemistry and biology?

Therefore, in this article, we summarize the latest research progress of different kinds of carbon nanomaterials in the fields of drug delivery and tissue engineering. Although the behavior of such materials *in vivo* has long been a concern, the advantages of carbon nanomaterials in biomedical applications are prominent. Regarding the three types of carbon nanomaterials discussed in this paper, including CDs, CNTs and graphene, each has unique physical and chemical properties. These properties are attributable to their specific chemical structure and nanometer size. The unique physicochemical properties of carbon nanomaterials make them suitable for the delivery of certain drugs and tissue engineering. Carbon nanomaterials can enable closer interdisciplinary links between nanotechnology, biology and medicine. They pave the way for biologists and clinicians to develop more powerful and useful tools.
